# 持久性肿瘤细胞的分子机制和靶向治疗研究进展

**DOI:** 10.3760/cma.j.issn.0253-2727.2023.08.018

**Published:** 2023-08

**Authors:** 想 李, 黎 王, 维莅 赵

**Affiliations:** 上海交通大学医学院附属瑞金医院，国家转化医学中心，医学基因组学国家重点实验室，上海血液学研究所，上海 200025 Ruijin Hospital Affiliated to Shanghai Jiao Tong University School of Medicine, National Research Center for Translational Medicine at Shanghai, State Key Laboratory of Medical Genomics, Shanghai Institute of Hematology, Shanghai 200025, China

在抗生素耐药的感染病灶中存在一类被称为持久性细菌的细菌亚群，以生长和代谢停滞作为主要机制以逃避抗生素的杀伤，若条件适合存活，持久性细菌将从暂时休眠状态中激活，感染可再次发作[1]。相似地，持久性肿瘤细胞指常规抗肿瘤治疗难以消除的一类肿瘤细胞亚群，这群细胞可通过进入一种可逆的缓慢增殖状态逃避化疗、放疗或某些靶向治疗导致的细胞凋亡，在抗肿瘤治疗中持续存活，形成化疗耐药储存库或微小残留病（MRD），介导抗癌治疗数年后肿瘤复发和转移[2]–[4]。

持久性肿瘤细胞以生长处于缓慢或停滞状态、细胞代谢高度灵活、细胞表型和肿瘤免疫微环境高度可塑性、对细胞凋亡抵抗、对铁死亡敏感等为特征[5]–[6]。有学者提出，休眠细胞、静止细胞、耐受细胞和持久细胞是癌症中同一靶点的4个同义词，均指常规抗癌治疗无法根除的肿瘤细胞亚群，且这4种状态下的肿瘤细胞相似地表现为细胞增殖缓慢、长期存活及干细胞样特点，均是促进肿瘤复发的关键因素[7]。因此，亟待深入探索介导肿瘤复发耐药的持久性肿瘤细胞生物学特征的分子机制，寻找特异、有效的靶向治疗新靶点，清除难以杀灭的肿瘤细胞，这对改善耐药和复发的肿瘤患者的生存具有重要的指导意义。本文将从持久性肿瘤细胞生物学特征的分子机制及相关靶向治疗策略等方面进行综述。

一、持久性肿瘤细胞生物学特征的分子机制

1. 增殖缓慢或停滞状态：组蛋白修饰作为表观遗传学修饰的重要组成部分，主要发生在其特定赖氨酸的非结构化N末端尾部，包括甲基化、乙酰化、磷酸化和SUMO化修饰等，特定基因启动子上这些修饰的组合形成了“组蛋白密码”，是转录抑制或激活的决定因素[8]。表观遗传学重编程在诱导和维持持久性肿瘤细胞缓慢增殖状态中发挥了重要作用。

据报道，第一代EGFR酪氨酸激酶抑制剂（TKI）厄洛替尼治疗后产生的非小细胞肺癌持久耐药肿瘤细胞亚群（DTPs）的染色质状态的维持需要IGF-1R信号传导和组蛋白去甲基化酶KDM5A的参与，使用IGF-1R抑制剂或靶向KDM5A可选择性消除DTPs[3]。近年来，已证实在TKI治疗后，脑胶质母细胞瘤干细胞可通过可逆地转变为缓慢细胞增殖或滞育样状态介导化疗耐药，此状态依赖KDM6参与的抑制性染色质的重分布和Notch通路的上调表达，DTPs对KDM6敲除均高度敏感，敲除KDM6B导致的生长抑制更为显著[9]。另外，Guler等[10]发现，DTPs存活还依赖于H3K9和H3K27甲基化增加的抑制性染色质状态的维持，在LINE-1元件中观察到H3K9me3的增加最为显著，抑制SETDB1和G9a，可破坏LINE-1元件抑制性染色质状态，减少DTPs，从而克服肿瘤耐药性。此外，研究表明，TET2可通过抑制TNF-α信号组分表达并阻碍其促凋亡信号传导控制慢循环状态肿瘤细胞的数量和生存，且由TET2产生的5hmC可标记这群细胞的基因组，作为癌症患者复发和生存的预测性生物标志物，故敲低TET2也可作为消除DTPs的潜在策略，延长患者生存[4]。

除表观遗传学重编程外，其他机制也参与维持DTPs缓慢增殖或滞育样表型。在结直肠癌持久细胞PDX模型及3D培养的乳腺癌持久细胞类器官模型中均观察到DTPs与滞育胚胎在基因表达谱上的相似性，特征是mTOR和Myc活性的降低，细胞自噬的增加[11]–[12]。据报道，一项研究通过活体遗传谱系追踪系统对单细胞进行纵向追踪，发现了在化疗前已存在休眠状态的LGR5^+^p27^+^结肠癌细胞，其在化疗期间持续存在，随后克隆扩增，转录组学提示COL17A1在这群休眠细胞中表达上调，通过敲除COL17A1可清除休眠LGR5^+^p27^+^癌细胞亚群，促进结肠癌MRD对化疗的敏感[13]。Álvarez-Varela等[14]揭示，高表达Mex3a的结直肠癌持久性细胞在化疗后立即下调WNT/干细胞基因程序，转变为类似YAP^+^胎儿肠道祖细胞的瞬时状态，并在化疗结束后再生为肿瘤细胞从而介导肿瘤复发，然而Mex3a缺陷细胞则分化为杯状细胞样表型，此表型对化疗敏感，因此Mex3a可作为根除结直肠DTPs的潜在靶点。

2. 细胞代谢高度灵活：与增殖肿瘤细胞Warburg效应不同，大多持久性肿瘤细胞具有以下共同代谢特征，即尽量减少葡萄糖消耗，积极利用其他能源，如脂肪酸等，并将能量代谢方式由糖酵解转变为线粒体氧化呼吸[5]。

研究发现，应用阿糖胞苷治疗后持续存活的急性髓系白血病（AML）细胞具有脂肪酸氧化（FAO）增加、CD36上调和高氧化磷酸化反应（OXPHOS）的特点，靶向线粒体蛋白合成、电子转移或脂肪酸氧化可诱导细胞向低OXPHOS转移，并显著增强阿糖胞苷的抗白血病作用[15]。真核生物脂肪酸氧化有两种方式：CPT1介导的线粒体脂肪酸氧化和ACOX1介导的过氧化物酶体脂肪酸氧化。据报道，PPARα-PGC1α-ACOX1轴介导的过氧化物酶体脂肪酸氧化是维持黑色素瘤中持久细胞存活的重要机制，这群细胞中CPT1和ACOX1的表达均上调，但只有沉默ACOX1会导致DTPs活性显著降低，且这些结果与高FAO黑色素瘤患者生存期短及BRAF/MEK抑制剂敏感患者肿瘤组织PPARα表达水平低等临床发现吻合[16]。

然而，持久细胞/静息细胞的主要代谢形式也可能因肿瘤类型不同而存在差异。研究发现，三阴性乳腺癌静息细胞可通过激活HIF通路、糖酵解通路等营造一个包含免疫抑制性成纤维细胞、功能失调的树突状细胞和高度耗竭性T细胞的缺氧性免疫抑制性微环境来抵抗T细胞的攻击，此细胞的代谢方式似与增殖肿瘤细胞Warburg效应相契合，但与大多数持久性肿瘤细胞的代谢特征存在差异[17]。此外，在儿童急性B淋巴细胞白血病（B-ALL）中，通过比较初诊、诱导治疗第19天和复发三个阶段白血病细胞的单细胞转录组数据，研究者发现残留白血病细胞的细胞周期多处于静息状态且低氧信号通路显著激活；另外，体内外实验模型也证实化疗药物处理后B-ALL残余细胞低氧信号通路的关键调控蛋白HIF-1α表达升高，下游信号通路激活，使用HIF-1α小分子抑制剂PX478可在体内外协同化疗药物有效杀伤B-ALL细胞系和原代B-ALL细胞，进而显著延长B-ALL PDX小鼠的生存时间[18]。

研究发现，化疗耐药AML干细胞（LSCs）mTOR通路被抑制但细胞自噬显著增强，亮氨酸可有效激活mTOR通路并抑制细胞自噬发生，小鼠体内研究证实亮氨酸阿霉素可有效清除LSCs，显著延长AML小鼠生存期，此药物可作为潜在的靶向细胞代谢的白血病治疗新药物[19]。

3. 细胞表型可塑性：细胞可塑性也称为表型转换，允许细胞采用不同表型以适应环境变化，持久性肿瘤细胞可通过细胞可塑性促进免疫逃逸并对抗肿瘤药物产生耐药性，主要机制为上皮-间充质转化（EMT）和药物诱导的转分化[20]。

EMT是上皮细胞失去其特性转换为间充质细胞并获得侵袭和转移能力的可逆过程，然而，EMT激活与肿瘤细胞的恶性特征有关，包括促进迁移和侵袭等[21]。研究发现，E-钙黏蛋白丢失、波形蛋白增加、Src通路和Hakai表达上调等与EMT促进DTPs存活密切相关[22]。

转分化，又称为谱系可塑性，被认为是介导肿瘤进展、耐药和复发的一种新机制，目前研究较多的是药物诱导的神经内分泌转分化。最近，有研究报道FOXA2可直接调控KIT通路的特异性激活从而驱动前列腺癌神经内分泌转分化过程，通过敲低FOXA2或用药物抑制KIT通路可显著抑制神经内分泌前列腺癌存活[23]。

4. 肿瘤微环境可塑性：肿瘤微环境由多种类型细胞在独特的代谢过程中形成，其中血管负责供应氧气和营养物质并清除废物，而基质细胞和免疫细胞通过分泌信号分子和细胞外基质成分调节肿瘤生长[24]，微环境中的某些特性对维持持久性肿瘤细胞存活具有重要意义。

癌症相关成纤维细胞（CAFs）是肿瘤微环境中丰度最高的细胞群，CAFs活化可激活许多致癌信号，促进癌细胞耐药性产生[25]。据报道，DTPs产生的乳酸增加可能是驱动CAFs通过NF-κB通路增加HGF分泌的关键因素，而CAFs分泌的HGF可与MET受体结合，激活PI3K/AKT和MAPK通路，从而驱动胶质母细胞瘤、结肠癌和黑色素瘤的耐药和复发[25]–[26]。另外，在胰腺导管腺癌中，通过shRNA沉默IRAK4或应用IL-1受体拮抗剂Anakinra拮抗IL-1β，可降低NF-κB通路活性，恢复持久性肿瘤细胞对吉西他滨的敏感性[27]。

除了CAFs，肿瘤相关巨噬细胞（TAMs）也是参与DTPs存活的重要角色，特别是具有抗炎M2样表型的TAMs，其通过分泌生长因子、抗炎细胞因子和免疫抑制因子等，驱动肿瘤进展和耐药。HER2抑制剂诱导的乳腺癌残留肿瘤细胞可通过TNF-α/NF-κB信号通路上调CCL5的表达，进而招募表达CCR5的TAMs，这些TAMs可通过胶原沉积的方式促进残余肿瘤细胞的持续存活[3]。另外，放疗抗性乳腺癌细胞中的HER2已被证明可通过NF-κB促进CD47表达，进而促进肿瘤细胞逃避巨噬细胞的吞噬，同时阻断HER2和CD47可有效清除放疗抗性乳腺癌细胞[28]。

在急性淋巴细胞白血病（ALL）中，化疗药物（如阿糖胞苷、柔红霉素、6-巯基嘌呤等）可激活ATM依赖性NF-κB通路，诱导GDF15、CCL3和CCL4等生态位保护相关细胞因子表达，从而募集骨髓基质细胞并促进骨髓保护性生态位形成，促进化疗残余细胞存活和耐药。阻断ATM依赖性NF-κB通路可使ALL对化疗药物敏感，为根除化疗耐药残留ALL细胞提供新策略[29]。

据报道，Galeano Niño等[30]利用原位空间分析技术、单细胞测序等方法揭示了肿瘤微生物与癌症持久性的潜在关系。通过分析口腔鳞状细胞癌和结直肠癌（CRC）样本，发现肿瘤相关细菌存在于高度免疫抑制微环境中，且细菌富集肿瘤区域呈低血管化和低增殖状态，细菌显性肿瘤细胞的基因表达特征为参与癌症进展信号通路上调，包括EMT、PI3K-AKT-mTOR、缺氧和干扰素反应通路等；另外，来源于CRC的具核梭杆菌可在细菌感染肿瘤部位诱导髓系细胞募集，促进CRC上皮细胞的转录变化，从而促进肿瘤细胞对周围环境的侵袭并赋予其静止特性。由此，细菌感染显性肿瘤细胞所表现出的特征与持久性肿瘤细胞有相似性，肿瘤微生物也可能参与诱导DTPs形成。

5. 细胞凋亡抵抗及铁死亡敏感：持久性肿瘤细胞倾向于逃避抗肿瘤药物诱导的细胞凋亡而存活，且具有铁死亡敏感的特征[31]。

既往研究认为，一旦细胞色素C释放到细胞质中，细胞凋亡就无法停止，但越来越多的研究显示，一些细胞可在此过程中存活。在最近的一项研究中，当肺癌细胞受到促凋亡药物作用时，被释放到细胞质中的细胞色素C可通过诱导HRI与ATF4合成，激活综合应激反应（ISR），当ISR的促生存作用超过细胞色素C诱导的细胞凋亡作用时，某些肿瘤细胞可因“凋亡失败”存活下来，成为持久性细胞，其中ATF4是应激反应的主要调节因子，可减少促凋亡蛋白和上调促生存基因，敲除或抑制ATF4和（或）HRI均可减少持久性细胞的产生[6]。据报道，选择性FLT3抑制剂奎扎替尼治疗后残余的AML细胞炎症基因上调，联合应用FLT3抑制剂和糖皮质激素（GC）可通过上调GC受体依赖性促凋亡蛋白BIM和降低GC受体依赖性抗凋亡蛋白MCL-1的表达，促进AML残留细胞的清除，预防FLT3突变型AML的MRD、突变耐药性和复发[32]。最近研究发现，使用新型HDAC抑制剂甲磺酸普依司他（PM）可显著抑制慢性髓性白血病（CML）干细胞存活的关键基因（如c-Myc、E2F、EZH2、Alox5、mTOR等），GLS1抑制剂联合PM可协同消除CML干细胞，为根治CML提供新的治疗策略[33]。

铁死亡是一种独特的由脂质过氧化物积累所致的铁依赖性非凋亡型细胞死亡形式，线粒体是活性氧的主要来源，持久性肿瘤细胞能量代谢向线粒体呼吸的转变使其更易暴露于氧化应激，因此DTPs具有铁死亡敏感性[3],[31]。研究表明，DTPs依赖GPX4生存，GPX4功能丧失可诱导DTPs发生铁死亡，并防止小鼠肿瘤复发，提示靶向GPX4可能是一种预防获得性耐药的治疗策略[34]。TP53突变是介导弥漫大B细胞淋巴瘤（DLBCL）耐药的预后不良基因突变。在TP53突变的细胞株及动物实验中发现，APR-246可诱导不同TP53突变型的DLBCL细胞发生铁死亡，为TP53突变的耐药DLBCL患者提供了新的潜在治疗途径[35]。

二、靶向清除持久性肿瘤细胞的策略

目前，研究者已提出许多分子机制解释肿瘤细胞的持久性表型，这些机制并不是独立存在的，而是相互作用、错综复杂的，如大多数持久性肿瘤细胞显示低Warburg效应，而三阴性乳腺癌静息细胞糖酵解通路上调，儿童B-ALL的MRD中缺氧通路上调，可用不同肿瘤的DTPs具有高度动态性和异质性解释。

特定的持久性表型为消除持久性肿瘤细胞提供了更精确的方案，目前靶向清除持久性肿瘤细胞的策略主要是通过应用CRISPR-Cas9、shRNA、siRNA及相关抑制剂等方法对持久性肿瘤细胞的关键基因或通路进行阻断，从而清除常规抗癌治疗后的持续存活细胞、预防病灶残留和肿瘤复发。靶向清除持久性肿瘤细胞的策略总结见表1。

**表1:** 靶向清除持久性肿瘤细胞的策略

肿瘤类型	耐受的治疗	促进肿瘤细胞持久性的机制	靶向清除持久性肿瘤细胞的策略
非小细胞肺癌[3]	厄洛替尼	增殖缓慢或停滞（表观遗传学重编程）	IGF-1受体抑制剂：AEW541；减低KDM5：shKDM5A；HDAC抑制剂：曲古抑菌素A
脑胶质母细胞瘤[9]	达沙替尼、克莱拉尼	增殖缓慢或停滞（表观遗传学重编程）	抑制Notch通路：Compound E（γ-分泌酶抑制剂）；抑制KDM6：CRISPR-Cas9敲除KDM6
非小细胞肺癌[10]	厄洛替尼	增殖缓慢或停滞（表观遗传学重编程）	抑制STDB1和G9a：shSTDB1；G9a抑制剂：UNC-0638
结直肠癌[4]	多西环素	增殖缓慢或停滞（表观遗传学重编程）	抑制TET2：CRISPR-Cas9敲低TET2
乳腺癌[12]	多西他赛或阿法替尼	增殖缓慢或停滞	CDK9抑制剂：NVP2、SNS032
结直肠癌[13]	氟尿嘧啶、奥沙利铂、伊立替康、曲妥珠单抗	增殖缓慢或停滞	抑制COL17A1：shCOL17A1
结直肠癌[14]	氟尿嘧啶、奥沙利铂、SN38	增殖缓慢或停滞	抑制Mex3a：FKBP-caspase-9敲除Mex3a
急性髓系白血病[15]	阿糖胞苷	细胞代谢适应性（脂肪酸氧化）	线粒体蛋白合成抑制剂：替加环素；电子传递链复合物Ⅰ抑制剂：二甲双胍；靶向脂肪酸氧化：乙莫克舍（CPT1抑制剂）
黑色素瘤[16]	BRAF抑制剂：PLX4720；MEK抑制剂：PD0325901	细胞代谢适应性（脂肪酸氧化）	抑制ACOX1：shACOX1
三阴性乳腺癌[17]	T细胞治疗	细胞代谢适应性（糖酵解）	抑制HIF-1α：CRISPR-Cas9敲除HIF-1α
儿童急性B淋巴细胞白血病[18]	CCCG-ALL-2015方案	细胞代谢适应性（低氧信号通路）	HIF-1α抑制剂：PX478
急性髓系白血病[19]	阿糖胞苷、阿霉素	细胞代谢适应性（自噬）	抑制细胞自噬：亮氨酸阿霉素
非小细胞肺癌[22]	吉非替尼或奥希替尼	细胞表型可塑性（上皮-间充质转化）	抑制Src通路/Hakai和Hakai/E-钙黏蛋白相互作用以逆转E-钙黏蛋白表达：JMF3086（HDAC和HMGR双重抑制剂）
前列腺腺癌[23]	雄激素去势治疗	细胞表型可塑性（转分化）	KIT抑制剂：伊马替尼、索拉非尼、舒尼替尼、卡博替尼
胰腺导管腺癌[27]	吉西他滨	微环境可塑性（癌症相关成纤维细胞）	抑制IRAK4：shIRAK4、AS2444697；抑制IL-1β：抗人IL-1β抗体、阿那白滞素（IL-1受体拮抗剂）
乳腺癌[28]	放疗	微环境可塑性（肿瘤相关巨噬细胞）	抗CD47抗体和HER2抑制剂：曲妥珠单抗
急性淋巴细胞白血病[29]	阿糖胞苷、柔红霉素、6-巯基嘌呤、甲氨蝶呤、长春新碱、环磷酰胺、地塞米松	微环境可塑性（骨髓保护性生态位）	阻断ATM依赖性NF-κB通路：Ku60019（ATM抑制剂）；NF-κB通路抑制剂：吡咯烷二硫代甲酸铵
肺癌[6]	BH3类似物：ABT-737；MCL-1抑制剂：S63845	细胞凋亡抵抗	阻断细胞色素C-HRI-ATF4通路：ISRIB（ISR抑制剂）、siATF4
急性髓系白血病[32]	FLT3抑制剂：奎扎替尼	细胞凋亡抵抗	上调糖皮质激素受体依赖性促凋亡蛋白BIM，降低糖皮质激素受体依赖性抗凋亡蛋白MCL-1：联合应用FLT3抑制剂和糖皮质激素
慢性粒细胞白血病[33]	伊马替尼	促进生存信号传导	HDAC抑制剂：甲磺酸普依司他；降低PM对CML干细胞谷氨酸代谢的增加作用：GLS1抑制剂：BPTES
乳腺癌[34]	拉帕替尼	铁死亡敏感	GPX4抑制剂：RSL3、ML210

三、总结

综上，持久性肿瘤细胞的生物学特征介导其在化疗、放疗及相关分子靶向治疗后持续存活，并形成MRD，最终导致肿瘤复发和临床进展。近年来，针对癌症持久性的机制研究虽已取得了巨大的进展，但也存在一定的缺陷，如相关研究大多局限于肺癌、乳腺癌、结直肠癌、黑色素瘤等实体瘤的范畴，而在血液系统肿瘤领域如淋巴瘤、多发性骨髓瘤中研究较少。另外，迄今为止仍未有一种靶向持久性肿瘤细胞的治疗策略成功转化为临床应用，可能是由于目前的治疗方法未整合出完善的持久性机制同时对抗这群细胞的代谢和增殖特征、动态可塑性及其与肿瘤微环境间复杂的相互作用等[5]。因此，迫切需要对持久性肿瘤细胞进行更深入、细致、全面的研究，制定更完善的靶向治疗策略，提高癌症治疗后复发耐药患者疗效，使更多患者获得治愈。
